# Structural analysis and blood-enriching effects comparison based on biological potency of Angelica sinensis polysaccharides

**DOI:** 10.3389/fphar.2024.1405342

**Published:** 2024-06-17

**Authors:** Yunxia Tian, Xiaorui Shen, Tingting Hu, Ziyu Liang, Yu Ding, Huilian Dai, Xinyuan Liu, Tulin Lu, Fangzhou Yin, Yachun Shu, Zhijun Guo, Lianlin Su, Lin Li

**Affiliations:** ^1^ School of Pharmacy, Nanjing University of Chinese Medicine, Nanjing, China; ^2^ Jiangsu Province Hospital of Chinese Medicine, Affiliated Hospital of Nanjing University of Chinese Medicine, Nanjing, China; ^3^ China Resources Sanjiu Pharmaceutical Co., Ltd., Shenzhen, China

**Keywords:** Angelica sinensis, polysaccharide, structure, biological potency, blood tonicity

## Abstract

Angelica sinensis is a long-standing medicine used by Chinese medical practitioners and well-known for its blood-tonic and blood-activating effects. Ferulic acid, ligustilide, and eugenol in Angelica sinensis activate the blood circulation; however, the material basis of their blood-tonic effects needs to be further investigated. In this study, five homogeneous Angelica sinensis polysaccharides were isolated, and their sugar content, molecular weight, monosaccharide composition, and infrared characteristics determined. Acetylphenylhydrazine (APH) and cyclophosphamide (CTX) were used as inducers to establish a blood deficiency model in mice, and organ indices, haematological and biochemical parameters were measured in mice. Results of *in vivo* hematopoietic activity showed that Angelica sinensis polysaccharide (APS) could elevate erythropoietin (EPO), granulocyte colony-stimulating factor (G-CSF), and interleukin-3 (IL-3) serum levels, reduce tumor necrosis factor-α (TNF-α) level in mice, and promote hematopoiesis in the body by regulating cytokine levels. Biological potency test results of the *in vitro* blood supplementation indicated strongest tonic activity for APS-H_2_O, and APS-0.4 has the weakest haemopoietic activity. The structures of APS-H_2_O and APS-0.4 were characterized, and the results showed that APS-H_2_O is an arabinogalactan glycan with a main chain consisting of α-1,3,5-Ara(f), α-1,5- Ara(f), β-1,4-Gal(p), and β-1,4-Gal(p)A, and two branched chains of β-t-Gal(p) and α-t-Glc(p) connected to each other in a (1→3) linkage to α-1,3,5-Ara(f) on the main chain. APS-0.4 is an acidic polysaccharide with galacturonic acid as the main chain, consisting of α-1,4-GalA, α-1,2-GalA, α-1,4-Gal, and β-1,4-Rha. In conclusion, APS-H_2_O can be used as a potential drug for blood replenishment in patients with blood deficiency, providing a basis for APS application in clinical treatment and health foods, as well as research and development of new polysaccharide-based drugs.

## 1 Introduction

Angelica sinensis is the dried root of the Umbelliferae plant Angelica sinensis (Oliv.) Diels, belonging to variety of Chinese medicinal herbs with the same source of food and medicine, which are mentioned in” Shen Nong’s Herbal Classic” and are revered as sacred remedies for blood-related conditions (Chen et al., 2019). Angelica sinensis contains various active ingredients and exhibits a broad spectrum of pharmacological effects, particularly known for its ability to fortify and stimulate blood circulation. Angelica sinensis has a clear material basis for activating blood circulation. Chemical components such as ferulic acid, ligustilide and eugenol demonstrate potent antiplatelet properties, contributing to reduced platelet aggregation and improved blood rheology (Zhu, 2019). However, the material basis of the blood tonic effect needs to be researched further.

Blood deficiency syndrome (BDS) is commonly treated in traditional Chinese medicine (TCM). It is a pathological state of blood deficiency and loss of nourishment of the internal organs and veins caused by excessive blood loss or injury to the spleen and stomach, resulting in insufficient blood in the middle jiao, insufficient sources of qi and blood biochemistry, and blood stagnation (Zhang et al., 2011). Blood deficiency characterized by blood reduction in quantity can be regarded as clinical anemia (Qian and Pan, 2018). Tumors, infections, drug side effects, and radiation therapy can also cause anemia symptoms. Anemia diagnostic indices are mainly hemoglobin concentration (HGB) and the number of red blood cells (RBC) lower than the normal standard (Huang, 2005). Hematopoietic growth in factors refers to a group of active proteins that facilitate the differentiation, proliferation, and directed maturation of hematopoietic cells in the bone marrow. These include IL-3, G-CSF, EPO, and thrombopoietin (TPO), which can regulate the proliferation and differentiation of blood cells and play a role in hematopoietic supplementation by promoting hematopoietic growth factor secretion. Clinically, anemia is mainly corrected by supplementing with iron, vitamin B_12_ and other drugs, but these may cause gastrointestinal discomfort, allergies, and other side effects. Many TCMs and compound formulas have the effect of tonifying and nourishing blood, such as, Angelica Sinensis (Liu et al., 2010), Radix Rehmanniae Preparata (Liu Y. Y. et al., 2022), Polygonati Rhizoma (Wang et al., 2022), Panax Notoginseng (Chen et al., 2023), Si Wu Tang (Liu Y. Y. et al., 2022), and Danggui-Buxue-Tang (Huang et al., 2016).

Polysaccharides exhibit extensive potential applications within the medical and nutraceutical sectors. Owing to their excellent biocompatibility, multifaceted bioactivities, abundant availability, minimal toxicological profiles, and capacity to serve as efficient drug delivery vehicles, polysaccharides are poised to emerge as prospective therapeutic agents or drug vectors for both the treatment and prophylaxis of various diseases. Currently, drugs made from astragalus, mushroom, poria, Ganoderma lucidum, and ginseng polysaccharides have been approved and marketed in China and are widely used in the fields of immune regulation and antitumor activity (Bai et al., 2021). APS is one of the main active ingredients of Angelica sinensis and has various pharmacological activities, including antitumor (Cao et al., 2010), antioxidant (Pan et al., 2018), immunomodulatory (Wang et al., 2016), hematopoietic (Lee et al., 2012), and hepatoprotective effects (Cao et al., 2018). Its physicochemical properties and biological activities are closely related to its monosaccharide composition, relative molecular mass, conformation, glycosidic bond position, and advanced conformation (Wang et al., 2011). APSs delay premature senescence of hematopoietic cells by down-regulating Wnt/β-catenin signaling (Niu et al., 2023). APS reverses D-gal-induced hematopoietic degeneration in a rat model of senescence by attenuating oxidative stress in the hematopoietic microenvironment (Jing et al., 2023). APS reverses CFA-induced anaemia in rats with arthritis by inhibiting the activation of IL-6/JAK2/STAT3 signalling pathway induced by complete Freund’s adjuvant (CFA) (Li et al., 2020). Among the various chemical components with blood replenishing effects in Angelica sinensis soup, the blood replenishing effect of APS was the most significant (Ning et al., 2002). Studies have shown that the active components of Angelica sinensis play have blood tonic effects (Liu et al., 2010), mainly APSs. Lee JG et al. (Lee et al., 2012). Isolated the highest hematopoietic activity of the F2 component of monosaccharides arabinose (Ara) (51.82%), fructose (Fru) (1.65%), galactose (Gal) (29.96%), glucose (Glc) (4.78%) and galacturonic acid (GalA) (14.80%), but the primary structure has not yet been fully analyzed, and the conformational relationship remains unclear.

Consequently, this study aimed to extract a series of homogeneous APSs to elucidate their physicochemical properties and structural characteristics. Furthermore, the research endeavored to delve into the hematopoietic effects of these polysaccharides, both *in vivo* and *in vitro*, to identify the main sites of hematopoiesis and their structures. Additionally, the study sought to investigate the possible relationship between their structural features and hematopoietic activities. These results provided a theoretical basis for the application of APSs in food and medicine, as well as research and development basis for new polysaccharide-based drugs.

## 2 Material and methods

### 2.1 Materials and chemicals

Fresh roots of Angelica sinensis were grown in Min County, Gansu Province, in July 2022 (Gansu, China). Human erythroleukemia cell line K562 was obtained from Nanjing Beijia Biotechnology Co., Ltd. (Beijing, China). Specific pathogen-free (SPF)-grade male Kunming mice were purchased from Scribes Biotechnology Co. Ltd. (Henan, China). Glucosamine (GlcN), Mannose (Man), rhamnose (Rha), glucuronic acid (GlcA), GalA, Glc, Gal, Ara, cyclophosphamide (CTX), acetylphenylhydrazine (APH), Hemin, macroporous adsorption resin D101, DEAE Sephadex A-25, and Sephadex G-100 were purchased from Yuanye Biotechnology Co., Ltd. (Shanghai, China). T-series dextran standards, T-1 (Mw = 1000), T-10 (Mw = 10,000), T-40 (Mw = 40,000), T-100 (Mw = 100,000), and T-500 (Mw = 500,000) were purchased from Solaybao Technology Co., Ltd. (Beijing, China). EPO, G-CSF, IL-3, and TNF-α enzyme-linked immunosorbent assay (ELISA) kits were purchased from Hunan Aifang Biotechnology Co., Ltd. (Hunan, China). RPMI 1640-fetal bovine serum (FBS) culture medium was purchased from Beijia Biotechnology Co., Ltd. (Nanjing, China). Cell proliferation and activity detection kit CCK-8 obtained from Biyuntian Biotechnology Co., Ltd (Shanghai, China) was used. dimethyl sulfoxide (DMSO) (Yifeixue Biotechnology Co., Ltd., Nanjing, China), trifluoroacetic acid (TFA) and 5-methyl-2-phenyl-1,2-dihydropyrazole-3-one (PMP) (Macklin Biochemical Technology Co., Ltd., Shanghai, China), and deuterium oxide (Innokai Technology Co. Ltd., Beijing, China) were used. All chemical reagents were of analytical grade.

### 2.2 Extraction isolation and purification of the polysaccharide

Extraction: Slices of Angelica sinensis were extracted using 12 volumes of pure water. The process was repeated twice for 1.5 h each time. The extracts were concentrated and then mixed with 95% ethanol to achieve an 80% alcohol content, and the mixture was left at 4°C for 24 h for alcohol precipitation. The precipitate was collected and then redissolved. Sevag reagent (chloroform:nbutanol = 4:1, the volume ratio of organic solvent to polysaccharide solution was 1:3) was added to remove the protein until the protein gel layer disappeared. The polysaccharide solution was collected; pigments on the upper layer were removed using a large pore adsorption resin, and the eluate was dialyzed with flowing distilled water.

Separation and purification: 0.5 g/mL of APS was firstly separated on a DEAE Sephadex A-25 (80 cm × 3 cm) column, and then eluted with distilled water, 0.1, 0.2, 0.3, and 0.4M NaCl aqueous solution. The flow rate was 1.0 mL/min, and the eluent was collected at 5 mL/tube after the flow rate stabilized. The polysaccharide content in the eluate was determined using the phenol-sulfuric acid method. The elution curve was plotted with the number of tubes as the horizontal coordinate and the absorbance as the vertical coordinate. The preliminary purified fractions were collected, dialyzed, concentrated, and lyophilized. Further purification was performed on a Sephadex G-100 column (80 cm × 2.5 cm) and eluted with distilled water. The purified fractions were named APS-H_2_O, APS-0.1, APS-0.2, APS-0.3, and APS-0.4, respectively.

### 2.3 Determination of the polysaccharide content

The total sugar content of each sample was determined using the phenol–sulfuric acid method.

### 2.4 Determination of the molecular weight

Waters liquid chromatograph with an evaporative light scattering detector (ELSD) (pipette temperature of 115°C, airflow rate of 3.2 L/min) was used with ultrapure water as the mobile phase and a TSK-GEL G3000PWXL column at a flow rate of 0.8 mL/min. The polysaccharide sample and the series of Dextran standard were weighed precisely, dissolved in distilled water and prepared into a 5 mg/mL solution. Retention times of the polysaccharides were measured as T500, T100, T40, T10, and T1 for each of the standards and plotted against the retention times with logMw to obtain standard curves. Retention time of the polysaccharide was measured and the molecular weight was calculated from the standard curve.

### 2.5 Analysis of the monosaccharide components

The monosaccharide composition of the polysaccharide samples was determined by complete acid hydrolysis combined with PMP precolumn derivatization (Li et al., 2015). 1 mL of 1 mg/mL polysaccharide sample solution was poured into a hydrolysis tube, and 1 mL 4M TFA was added. N_2_ was used to remove air inside the tube and sealed, followed by hydrolysis in an oven at 110°C for 8 h. After the TFA was removed, 1 mL 0.6M NaOH solution was added to the tube and mixed well. Then 2 mL of 0.5 M PMP methanol solution was added to the mixture, vortexed to mix well, and reacted in a water bath at 70°C for 100 min. After cooling to room temperature, 2 mL of 0.3M HCl was added to neutralize and the mixture was spin-dried under reduced pressure. The extraction solution (chloroform: water = 1:1) was added to extract three times and the aqueous phase was passed through a 0.22 μm filter membrane on the machine to measure. Eight standard monosaccharides were subjected to the same treatment. The mobile phases were ammonium acetate aqueous solution (100 mmol/L, pH5.1) and acetonitrile (81:19, v/v) at a flow rate of 1.0 mL/min and detection wavelength of 250 nm.

### 2.6 Fourier transform-infrared (FT-IR) spectroscopy

The samples were prepared by KBr compression method and analyzed by Fourier transforminfrared spectrometer scanning in the wavelength range of 4000–400 cm^-1^ to determine the charact-eristic absorption peaks of each polysaccharide sample.

### 2.7 Methylation analysis

In this experiment, the methylation analysis of polysaccharide samples was performed by the Needs method. Briefly, polysaccharides were reduced with carboxymethyl cellulose (CMC) reagent, dried, and methylated by adding anhydrous DMSO, NaOH, and CH_3_I; this procedure was repeated until the broad peaks around 3400 cm^-1^ in the IR spectrum of the samples disappeared. Then formic acid and TFA were added for hydrolysis, spin-dried and reduced by adding 0.5M NaBH_4_ dilute base solution, acetylated by adding acetic anhydride:pyridine (1:1), spin-dried and extracted with extraction solution (chloroform:water = 1:1), and the organic phase was taken for on-line detection.

Gas chromatography–mass spectrometry (GC-MS) conditions:

Column: Agilent HP-5MS quartz capillary column (30 mm × 250 um×0.25 um).

Program warming: start from 100°C, keep 3 min, 20°C per minute, rise to 200°C and keep continuing for 5 min, 3°C per minute, rise to 230°C and keep continuing for 2 min, 10°C per minute, rise to 280°C and keep continuing for 8 min.

Inlet temperature: 200°C

Ion source EI: 230°C 70 eV

Carrier gas: N_2_

Carrier gas flow rate: 1 mL/min

Mass spectral scanning range (m/z): 40–400

### 2.8 Nuclear magnetic resonance (NMR) spectroscopy

Fifty milligrams (50 mg) of APS-H_2_O and APS-0.4 samples were each taken, fully dried, and 1 mL of heavy water added to them. They were then freeze-dried for 48 h, replaced three times, and 0.5 mL of heavy water was added and transferred to HMBC NMR tubes for one-dimensional ^1^H-NMR, ^13^C-NMR, and two-dimensional ^1^H–^1^H COSY, HSQC, HMBC, and NOESY spectroscopic analyses.

### 2.9 Evaluation of blood tonifying efficacy *in vivo*

The test animals were 6-week-old SPF-grade male Kunming mice, weighing 18–22 g, purchased from Henan Skebes Biotechnology Co. Ltd. (Certificate No.: SCXK (Yu) 2020-0005). After being acclimatized to the environment for 7 days, the mice were divided into six groups of 10 mice each: normal control (NC), blood deficiency model (MOD), positive control (FEJ, 20 mL/kg), APS low dose (APS-L, 100 mg/kg), APS medium dose (APS-M, 200 mg/kg), and APS high dose (APS-H, 400 mg/kg) groups. NC and MOD groups were given equal amount of distilled water by gavage at the same time of the day for nine consecutive days. Except for the NC group, all other groups were injected subcutaneously with APH saline solution (at doses of 20 mg/kg and 40 mg/kg, respectively) on the 2nd and 5th days of administration, and CTX saline solution (40 mg/kg) was injected intraperitoneally every day for four consecutive days from the 5th day onwards (He et al., 2012). The CN group was injected with an equal volume of saline solution.

#### 2.9.1 Body weight gain

The body mass of each group of mice on the first and last day of the test was determined, and the formula for calculating the change in body mass was: change in body mass (g) = body mass of mice (d10) - body mass of mice (d1).

#### 2.9.2 Organ index

The liver, spleen and thymus were dissected with scissors and rinsed with 4 °C saline to remove blood stains. Surface fat and connective tissues of the organs were then stripped clean, the water was absorbed by filter paper, and the mass of spleen and thymus were weighed. The thymus and spleen indices were obtained according to the following formulas: thymus index = thymus weight (mg)/rat weight (g) × 10 and spleen index = spleen weight (mg)/rat weight (g)*10.

#### 2.9.3 Blood routine test

In each group, blood was pre-diluted using EDTA anticoagulated blood collection tubes 24 h after the last drug administration and subjected to routine blood tests with an automatic blood analyzer. The calculation was performed to determine the number of RBC, white blood cells (WBC), HGB, hematocrit (HCT), and platelet (PLT) content in the whole blood of each group of mice.

#### 2.9.4 Changes in serum biochemical indicators in mice

The blood was allowed to stand at room temperature for 30 min, then the samples were centrifuged at 3000 rpm for 15 min, and the upper layer of serum was used for the biochemical indexes. The methods for the determination of EPO, G-CSF, IL-3, and TNF-α were strictly in accordance with the analysis kits manufacturers’ instructions.

### 2.10 Determination of blood supplementation potency *in vitro*

#### 2.10.1 Cell lines and culture

Human myeloid leukemia K562 cell line was selected and cultured in RPMI 1640 medium containing 10% fetal bovine serum in a cell culture incubator at 37°C, 5% CO_2_, and fully saturated humidity. The cells were passaged every 2–3 days. Logarithmic growth phase cells were used for the experiment.

#### 2.10.2 Blood supplementation potency determination

Blood replenishment potency of each APS was measured by the method of blood replenishment bioefficacy of Angelica sinensis tablets established by the research group in the early stages of the study. The hemoglobin level of each APS was repeated, and the effects of different concentrations of the standard and the test material on the relative hemoglobin content were analyzed. The concentration range with significant and quantity effect correlation was selected, and the blood replenishment potency of each APS was calculated according to formula 1 within this range (Liu H. H. et al., 2022).

PT=A*ds3dt3*2.51^T1+T2+T3‐S1‐S2‐S3T3‐T1+S3‐S1 (1)

Note: The dose groups d1, d2, and d3 of the standard substance (S) and test substance (T) corresponded to low, medium, and high doses, respectively, and the ratio between the two doses was 1:0.5.

## 3 Results

### 3.1 Isolation and purification of APS

**FIGURE 1:**
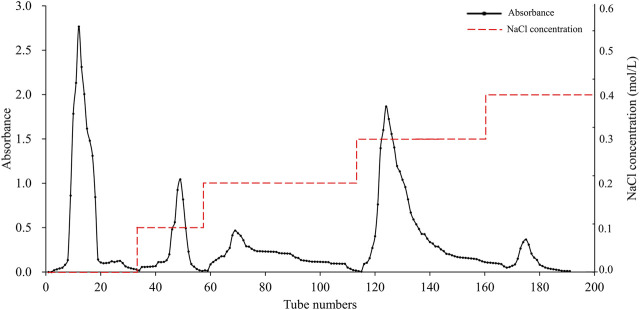
Purification of APS on DEAE Sephadex A-25 column.

The elution profile of APS on a DEAE Sephadex A-25 is shown in Figure 1, which was sequentially eluted with distilled water and solutions of 0.1, 0.2, 0.3, and 0.4 M NaCl. Each fraction was sampled separately on a Sephadex G-100 column for further purification, resulting in five pure polysaccharide fractions. The elution profile of these fractions is shown in Supplementary Figure S1. These samples were named APS-H_2_O, APS-0.1, APS-0.2, APS-0.3 and APS-0.4.

### 3.2 The content of APS

The standard curve equation for Glc based on ultraviolet-visible (UV-Vis) spectrophotometry was y = 0.0114x+0.0256 (*R* ^2^ = 0.9991), and the sugar contents of APS-H_2_O, APS-0.1, APS-0.2, APS-0.3 and APS-0.4 were measured to be 91.82, 93.05, 90.25, 89.19, and 92%, respectively.

### 3.3 Determination of the molecular weight

The purity and molecular weight of polysaccharides are prerequisites for studying their properties. In this experiment, the molecular weight of each APS was determined by the HPSEC-ELSD method, and the standard curve equation was y = −0.5493x+8.9252 (*R* ^2^ = 0.9975). The results of the HPSEC-ELSD determination are shown in Supplementary Figure S2. Each APS sample showed a single peak on the HPLC chromatogram and was a homogeneous polysaccharide. The molecular weights of APS-H_2_O, APS-0.1, APS-0.2, APS-0.3, APS-0.4 were 6.02×10^5^, 4.27×10^5^, 1.47×10^5^, 1.25×10^5^, and 1.37×10^5^, respectively.

### 3.4 Monosaccharide composition

**FIGURE 2:**
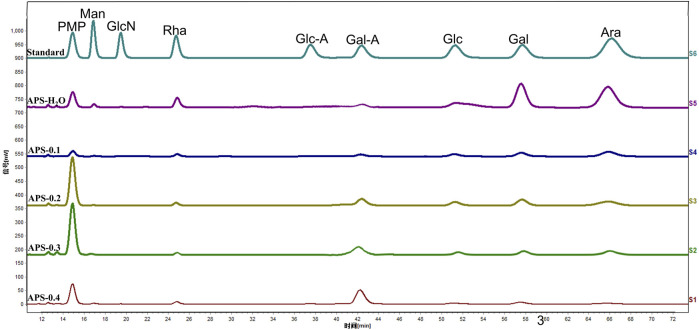
Monosaccharide composition of polysaccharides.

The composition of the APS monosaccharides varied with the different extraction processes, with the results depicted in Figure 2. APS-H_2_O, APS-0.1, APS-0.2, and APS-0.3, all consisted of Man, Rha, GalA, Glc, Gal, and Ara; whereas APS-0.4 contains no Ara and is mainly composed of Gal A. Some differences in the content of each monosaccharide were observed, reflected in the different molar ratios of the monosaccharides. Ara content gradually decreased with increasing eluent polarity in the DEAE column. The peak area of the derivatives of each polysaccharide after complete acid hydrolysis was substituted into the standard curve to determine the concentration of each monosaccharide and obtain the molar ratio between the monosaccharide components in the polysaccharide. The molar ratios are as follows:

APS-H_2_O: Man:Rha: GalA: Glu: Gal: Ara = 0.23:1.00:1.22:0.39:4.83:6.21;

APS-0.1: Man:Rha: GalA: Glu: Gal: Ara = 0.17:1.00:1.18:1.53:2.88:5.19;

APS-0.2: Man:Rha: GalA: Glu: Gal: Ara = 0.09:1.00:3.50:2.30:4.13:4.25;

APS-0.3: Man:Rha: GalA: Glu: Gal: Ara = 0.12:1.00:5.43:0.92:2.24:3.31;

APS-0.4: Man:Rha: GalA: Glu: Gal = 0.20:1.00:9.88:0.59:1.39.

### 3.5 FT-IR analysis

**FIGURE 3:**
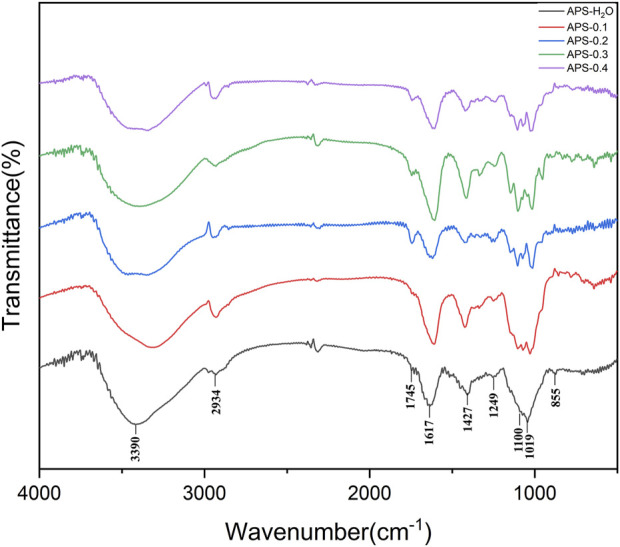
FT-IR spectrum of APS-H_2_O, APS-0.1, APS-0.2, APS-0.3 and APS-0.4.

The infrared scanning results for each polysaccharide sample are shown in Figure 3. The infrared scanning results of APS-H_2_O, APS-0.1, APS-0.2, APS-0.3, APS-0.4 are similar, with similar peak positions and typical absorption peaks of polysaccharides. Broad peak of 3700–3100 cm^-1^ is the stretching vibration peak of the -OH functional group of the polysaccharide samples, and the peak at 3000–2900 cm^-1^ was the stretching vibration absorption peak of C-H (Cao et al., 2006), which was weaker. The peak at 2900 cm^-1^ is the C-H stretching vibration absorption peak, which is weak because of the easy moisture absorption by the polysaccharide sample. The signal near 1613 cm^-1^ is the O-H deformation vibration produced by polysaccharides containing bound water (Liu et al., 2019). The peak at 1745 cm^-1^ is the absorption peak caused by the stretching vibration of C=O (-COOH or -CHO functional group) (Zhang et al., 2016), and the absorption peaks of polysaccharides are different in strength, which means that the peaks are different from one another. The absorption peaks near 1427 cm^-1^ were C-H bending vibration peaks (Li et al., 2020). the three peaks 1019, 1100, and 1249 cm^-1^ in the range of 1300–1000 cm^-1^ were C-O stretching vibration peaks of pyranose, indicating the existence of the pyranose ring. the peaks in the range of 880–830 cm^-1^ indicated the existence of α-glycosidic bonds (Kong et al., 2015). and the peaks near 770 cm^-1^ were D-O (-COOH or -CHO functional groups) absorption peaks, indicating the existence of different polysaccharides. The peak near 770 cm^-1^ is the symmetric telescopic vibration of the D-glucopyranose ring, indicating the presence of the pyranose ring structure of the polysaccharide (Wang et al., 2016).

### 3.6 Methylation analysis

After hydrolysis, reduction and acetylation of APS-H_2_O, the corresponding partially methylated alditol acetate (PMAA) was obtained, and the total ion flow chromatograms obtained by GC-MS detection and analysis are shown in Supplementary Figure S3. The GC chromatogram of APS-H_2_O showed nine major peaks, which were combined with the tandem mass spectrometry results of the characteristic fragments, the monosaccharide composition data and the PMAA standard database of the Centre for the Study of Complex Carbohydrates, Georgia University, United States (http://www.ccrc.uga.edu/specdb/ms/pmaa-/pframe.html). The nine peaks were identified as: t-Glc(p); 3-Ara(f); 5-Ara(f); t-Gal(p); 3,5-Ara(f); 2,4-Rha(p); 4-Gal(p); 6-Gal(p); 3,6-Glc(p). The ratio between each sugar residue was calculated based on the peak area of PMAA on GC with the corresponding response factor. The above results are summarised in Table 1. The major bond types in APS-H_2_O were 1,3,5-Ara(f), 1,5- Ara(f), 1,4-Gal(p), and t-Glc(p), with proportions of 30.64, 24.49, 14.00, and 11.72%, respectively. We hypothesized that APS-H_2_O has a main-chain structure consisting of 1,3,5-Ara(f), 1,5- Ara(f), and 1,4-Gal(p).

**TABLE 1:** Methylate analysis data of APS-H_2_O.

Monosaccharidc	Retention time (min)	Type of linkage	Molar ratio (%)	Mass fragments
Gal	9.889	t-Gal(p)	8.05	57,71,87,101,117,129,145,161,162,205
	10.729	4-Gal(p)	14.00	58,71,87,99,113,117,131,142,157,173,203,233
	11.051	6-Gal(p)	1.80	71,87,99,117,129,161,162,173.189,233
Ara	8.993	3-Ara(f)	2.06	58,71,87,99,117,129,233
	9.287	5- Ara(f)	24.49	58,71,87,101,117,129,173,189
	10.078	3,5-Ara(f)	30.64	58,85,99,117,127,141,159,201,261
Rha	10.207	2,4-Rha(p)	4.30	57,87,101,129,143,189,203,231
Glc	8.266	t-Glc(p)	11.72	58,71,87,102,117,129,189,161,162,205
	12.754	3,6-Glc(p)	2.94	57,71,87,117,129,139,189,205,233,245,305

Relative molar ratio (%) = Residual sugar peak area/Total peak area.

### 3.7 NMR spectrum analysis

**FIGURE 4:**
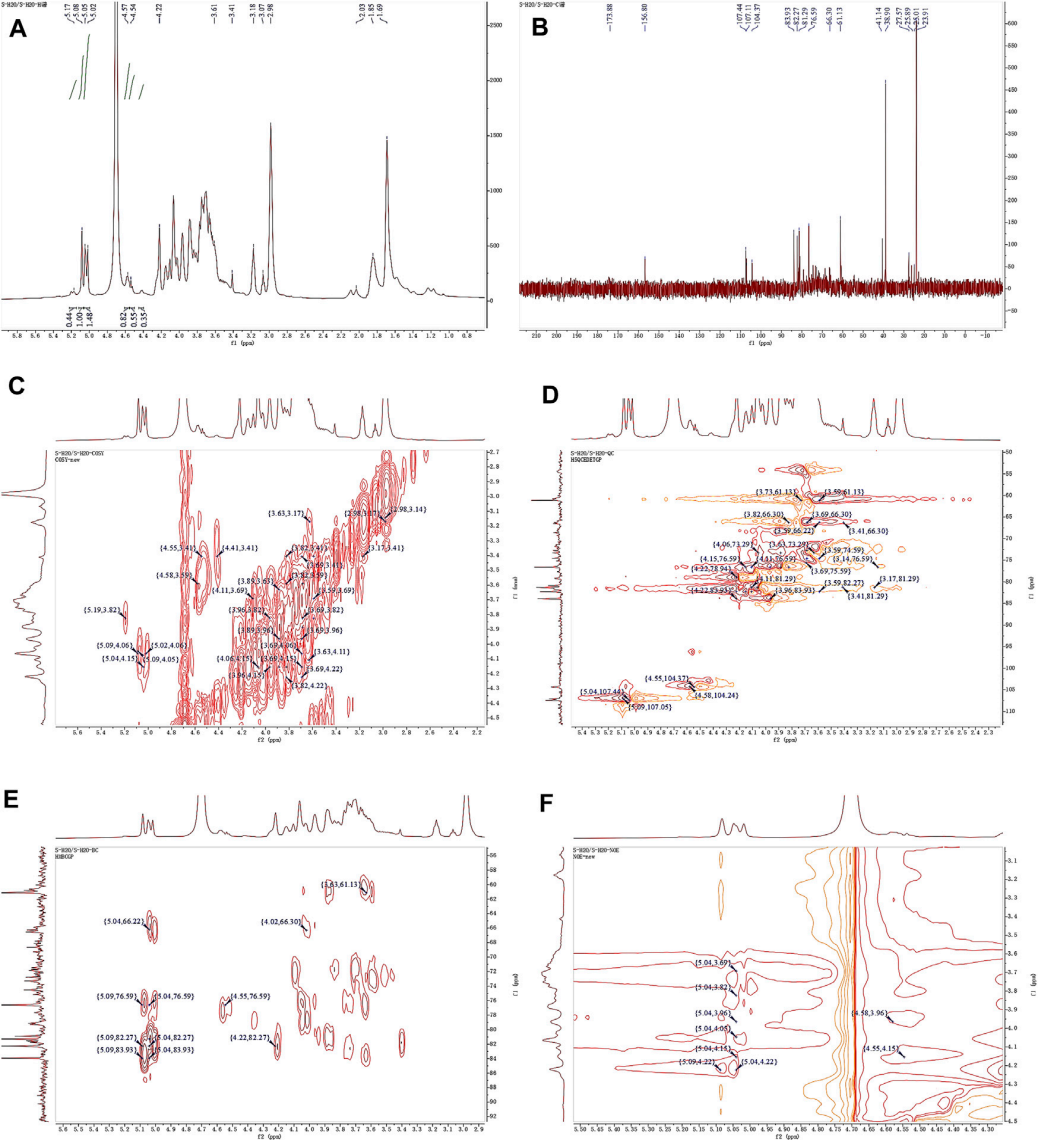
1D and 2D NMR spectra of APS-H_2_O. **(A)** ^1^H NMR. **(B)** ^13^C NMR. **(C)** COSY. **(D)** HSQC. **(E)** HMBC. **(F)** NOESY.

Observing the ^1^H NMR spectrum of APS-H_2_O (Figure 4A), heterohead hydrogen signals of 5.17, 5.08, 5.05, 4.57, 4.54 ppm were found in the heterogeneous head region (4.3–5.5 ppm);^13^C NMR spectrum (Figure 4B), carbonyl carbon signal of glucuronic acid (173.88 ppm) was found within 150–220 ppm, attributed to Gal carbonyl carbon signal of C6 of glyoxalate. The heterocarbon signals were 107.44, 107.11, and 104.37 ppm, and the signal in the range of 55–66 ppm was the exocyclic methylene-CH_2_-proton signal of the residual sugar pyran or furan ring, which in combination with the composition of the residual sugar, was attributed to C6 of the Gal residual sugar at 61.13 ppm.

In the HSQC spectrum (Figure 4D), the H/C correlation signals observed in the heterogeneous head region were indicative of specific monosaccharide compositions and methylated residual sugar types. These signals, with chemical shifts of 5.09/107.05, 5.04/107.44, 4.58/104.24, and 4.55/104.37 ppm, respectively, Based on the magnitude of the heterohead hydrogen intensity of the hydrogen spectra, the residual sugar corresponded to the probable residual sugar attributed to them in order α-1,3,5- Ara(f), α-1,5-Ara(f), β-1,4-Gal(p), β-t-Gal(p). After 5.04 ppm of isocaprotic hydrogen was attributed to isocaprotic hydrogen H1 in the α-1,5-Ara(f) residual sugar, the signal associated with it was found to be 4.15 ppm based on the ^1^H–^1^H COSY spectrogram (Figure 4C), confirming the position of H2 as 4.15 ppm, which, in combination with the HSQC spectrogram, confirms that the signal of H2/C2 is 4.15/76.59. Using this method, we continued to detect signals for H3/C3, H4/C4, and H5/C5 at 3.96/76.59, 3.82/66.30, and 4.22/82.27 ppm. Using the same methodology, the possible attributions of each position of the four main residual sugars, α-1,3,5-Ara(f)、α-1,5-Ara(f), β-1,4-Gal(p), and β-t-Gal(p) were determined (Table 2).

**TABLE 2:** Chemical shift assignment of glycosidic linkages in APS-H_2_O and APS-0.4.

Residues linkage	H1/C1	H2/C2	H3/C3	H4/C4	H5/C5	H6/C6
APS-H_2_O						
α-1,5-Ara(*f*)	5.04/107.44	4.15/76.59	3.96/76.59	3.82/66.30	4.22/82.27	-
α-1,3,5-Ara(*f*)	5.09/107.05	4.06/73.29	4.15/82.27	3.89/76.59	3.96/83.93	-
β-1,4-Gal(*p*)	4.58/104.24	3.59/74.59	3.69/75.59	4.11/81.29	3.63/73.29	3.59/61.13
β-t-Gal(*p*)	4.55/104.37	3.41/81.29	3.63/73.29	3.89/73.29	3.69/72.75	3.73/61.13
APS-0.4						
α-1,2-GalA(p)	5.73/106.80	4.20/78.29	3.65/72.90	3.91/68.14	4.02/74.19	-/169.42
α-1,4-Gal(p)	5.27/91.95	3.72/68.06	3.39/71.29	4.52/78.71	3.91/68.14	3.41/59.99
α-1,4-GalA(p)	5.03/99.03	3.65/70.00	3.89/68.38	4.50/78.71	3.72/68.06	-/170.71
β-1,4-Rha(p)	4.53/96.00	3.40/71.29	3.69/72.28	4.30/79.04	3.72/53.53	1.18/16.09

−, not detected.

In the nuclear overhauser effect spectroscopy (NOESY) spectra (Figure 4F), in the region of heterocapital hydrogens of the residual sugar, α-1,5-Ara(f), three signals were found as: 5.04/4.15, 5.04/3.82, and 5.04/4.22, which identified the Ara residual sugar as a furan-type residual sugar and the presence of a 1→5 glycosidic bond linkage. Similarly, the correlation signal of residual sugar α-1,3,5-Ara(f) was identified as 5.09/4.22, confirming the presence of a 1,5 linkage to residual sugar α-1,5-Ara(f) with the presence of a glycosidic bond. The NOESY signals of 4.55/4.15 for the residual sugar β-t-Gal(p) confirmed the existence of a 1,3-linked glycosidic bond with the residual sugar α-1,3,5-Ara(f), which is presumed to be a constituent part of the side chain. In the heteronuclear multiple bond correlation (HMBC) spectra (Figure 4E), in the α-Ara(f) residual sugar, the isocaprotic hydrogen correlation signals were 5.04/76.59, 5.04/82.27, indicating that the heterohead hydrogen of this residual sugar is remotely correlated with C2 in the same ring as well as another C5 of the same residue, confirming the glycosidic bond connecting 1→5 of the α-1,5-Ara(f) residual sugar in NOESY. In the α-1,3,5-Ara(f) residue sugar, a correlation of 5.09/83.93 confirmed the glycosidic bond connecting this residue to the same residue 1→5 of the α-1,3,5-Ara(f) residue sugar; and a correlation of 5.09/82.27 confirmed the glycosidic bond connecting this residue to the same residue 1→5 of the α-1,5-Ara(f) residue sugar.

Taken together, based on the methylated structure combined with the NMR data, APS-H_2_O was determined to be an arabinogalactan glycan, and a possible structure of the repeating unit in APS-H_2_O was hypothesized, as displayed in Figure 6.

**FIGURE 5:**
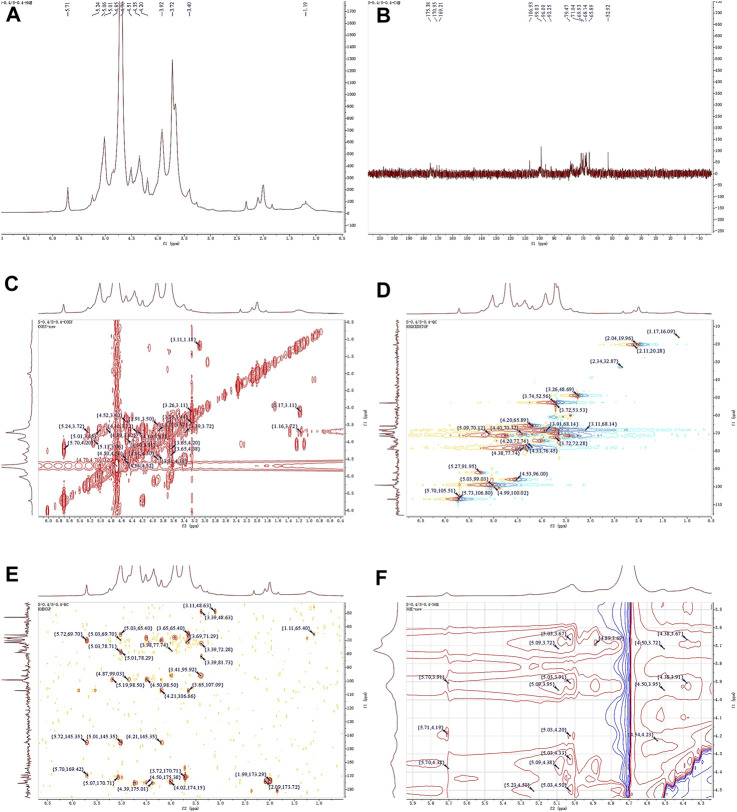
1D and 2D NMR spectra of APS-0.4. **(A)** ^1^H NMR. **(B)** ^13^C NMR. **(C)** COSY. **(D)** HSQC. **(E)** HMBC. **(F)** NOESY.

The ^1^H NMR spectrum of APS-0.4 (Figure 5A) showed that the isohead hydrogen signals were at 5.71, 5.24, 5.01, 4.85, 4.51, 4.35 ppm ^13^C NMR spectrum (Figure 5B), three carbonyl carbon signals for saccharide carbonyls were found in the range of 150–220 ppm: 175.38, 170.95, and 169.21 ppm, which indicated that APS-0.4 is acidic and has more acidic residual sugars. The carbon signals in the heterocarbon region were 106.93, 99.03, 96.00, and 92.25 ppm, and the H/C correlation signals in the heterocarbon region were 5.73/106.80, 5.27/91.95, 5.03/99.03, and 4.53/96.00 ppm in the heteronuclear single quantum coherence (HSQC) spectra (Figure 5D), which were assigned to the following residue sugars in the order of the composition of the monosaccharides and the heterocarbon intensities in the hydrogen spectra. The possible residual sugars corresponded to α-1,2-GalA(p), α-1,4-Gal(p), α-1,4-GalA(p), β-1,4-Rha(p) (Yao et al., 2021). Combined with the ^1^H–^1^H COSY spectra (Figure 5C), the possible attributes of each position of the four major residual sugars were determined, as shown in Table 2.

In the NOESY spectra (Figure 5F), multiple signals were found in the heterogeneous hydrogen region of the major residual sugar, α-1,4-GalA(p): 5.03/3.67, 5.03/3.91, 5.03/4.20, 5.03/4.50, suggesting that this hemi lactic acid residue sugar has a 1→4 glycosidic linkage and a 1→2 glycosidic linkage with residual sugar α-1,2-GalA(p); a correlation signal at 5.73/4.19 confirms the existence of a 1→2 glycosidic linkage within residual sugar α-1,2-GalA(p) itself; and the presence of a NOESY at 5.27/4.50 for residual sugar α-1,4-Gal(p) confirmed the presence of a NOESY at 5.27/4.50 for residual sugar α-1,4-GalA(p). signal at 5.27/4.50 confirmed the existence of a 1→4 glycosidic linkage of residual sugar α-1,4-Gal(p), whereas the signal at 4.54/4.50 may be the existence of a 1→4 glycosidic linkage of residual sugar β-1,4-Rha(p) to α-1,4-GalA(p). In the HMBC spectra (Figure 5E), the presence of iso-capsule hydrogens in Gal residual sugar α-1,4-GalA(p) The correlation signal of 5.03/78.71 confirmed a 1→4 glycosidic bond to this residual sugar itself, corroborating the NOESY analysis; 5.03/78.29 is a 1→2 glycosidic bond linkage with residual sugar α-1,2-GalA(p).

**FIGURE 6:**
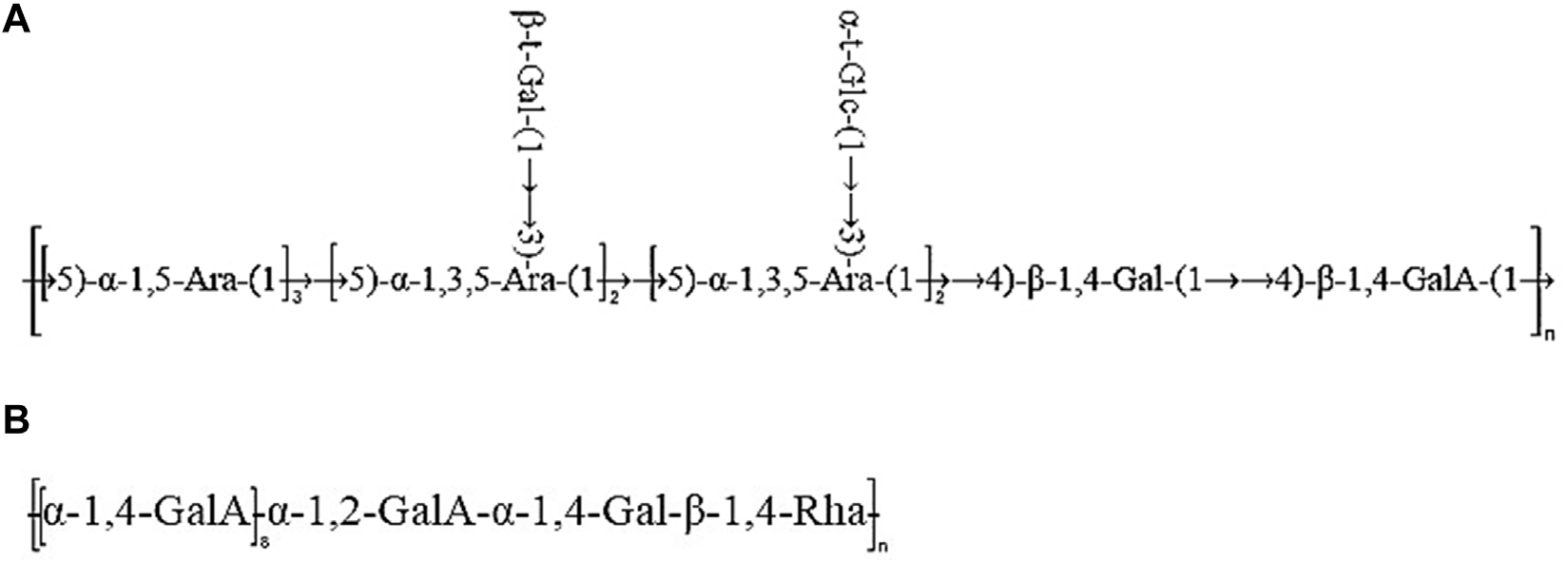
Predicted structure of APS-H_2_O **(A)** and APS-0.4 **(B)**.

Taken together, these analyses and literature studies indicate that APS-0.4 has GalA as the main chain (Cheng and Neiss, 2012), which may be pectinic in structure, suggesting a possible structure of the repeating unit in APS-0.4, as shown in Figure 6.

### 3.8 Evaluation of blood tonifying efficacy *in vivo*

#### 3.8.1 Body weight gain (BWG), spleen index (SI), and thymus index (TI)

The effects of APS on body weight and splenic gland index in model mice are shown in Table 3. Compared with the NC group, body weight gain (BWG) value and the thymus index (TI), were significantly reduced (*p* < 0.01), and the spleen index (SI) was significantly increased (*p* < 0.01) in MOD group. Compared with the MOD group, BWG value of the APS-H group increased significantly (*p* < 0.01), TI of mice in the APS-M and APS-H groups, was significantly higher (*p* < 0.01), and SI of mice in the APS-M and APS-H groups was significantly lower (*p* < 0.01).

**TABLE 3:** Effect of APS on ratio of BWG, SI and TI.

Groups	BWG/(g)	SI(mg/10 g)	TI (mg/10 g)
NC	7.65 ± 1.28	0.34 ± 0.09	0.29 ± 0.04
MOD	3.55 ± 1.68##	0.62 ± 0.09##	0.09 ± 0.02##
FEJ	7.52 ± 1.42**	0.34 ± 0.08**	0.25 ± 0.04**
APS-L	5.12 ± 1.31	0.51 ± 0.05	0.13 ± 0.02
APS-M	5.78 ± 1.41	0.47 ± 0.09**	0.17 ± 0.05**
APS-H	6.32 ± 2.02**	0.46 ± 0.10**	0.21 ± 0.02**

Note: Values given are the means ± SD, with n = 10. ##*p* < 0.01 compared to the NC, group. *p < 0.05, **p < 0.01 compared to the MOD, group.

#### 3.8.2 Blood routine test

**FIGURE 7:**
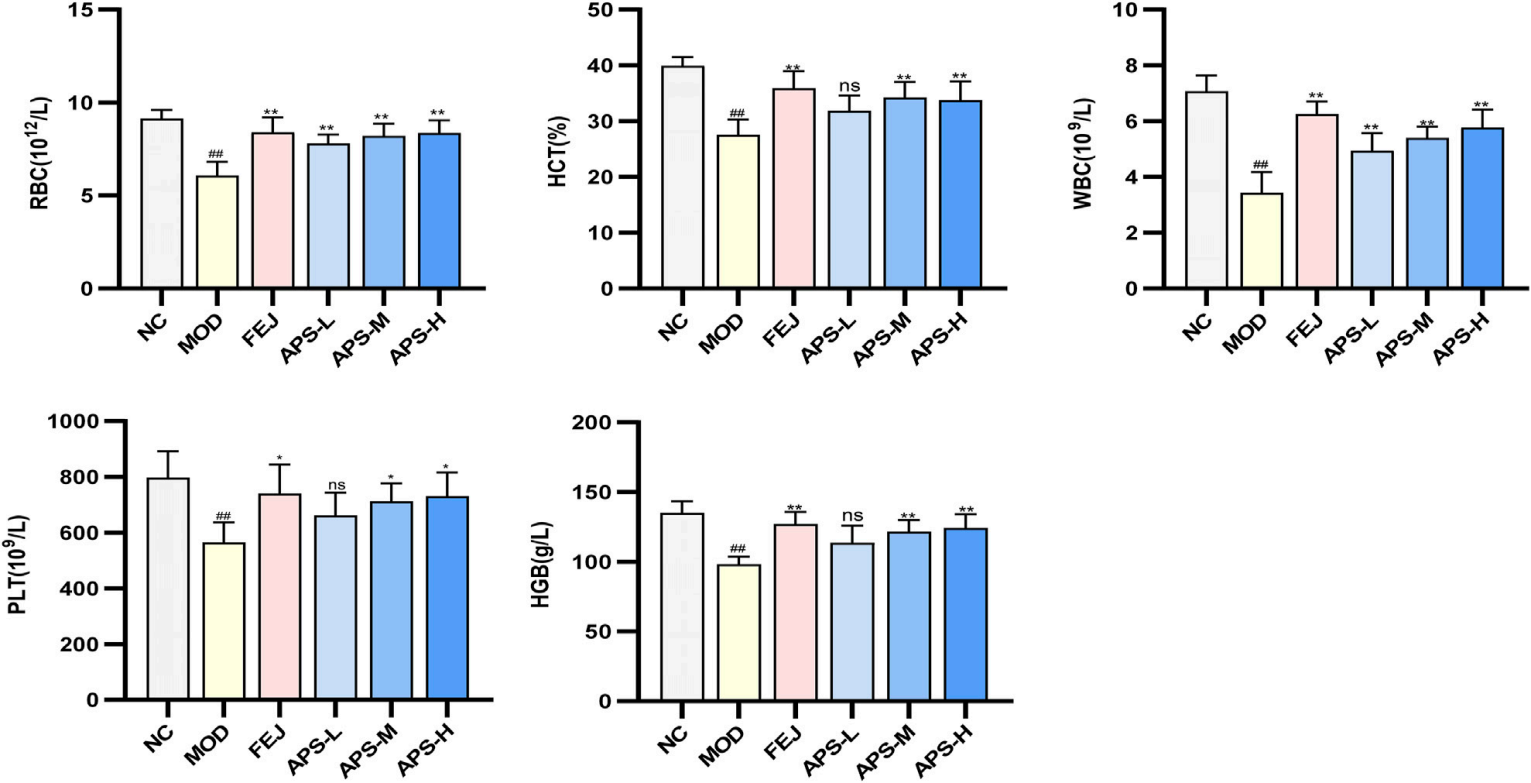
Effects of APS on blood routine in BDS mice.

The results of the routine blood tests are shown in Figure 7. Compared with the NC group, the levels of WBC, RBC, HGB, HCT, and PLT in the peripheral blood of mice in the MOD group were significantly lower (*p* < 0.01), indicating that the model of blood deficiency was successfully established. Compared with the MOD group, the content of RBC in the blood of mice in all administered groups was significantly higher (*p* < 0.01), and the levels of WBC, HGB, HCT, and PLT in the blood of mice in all drug groups except APS-L were significantly higher (*p* < 0.05, *p* < 0.01). These results indicated that APSs could effectively reverse blood deficiency induced by APH and CTX in mice.

#### 3.8.3 Analysis of EPO, G-CSF, IL-3, and TNF-α in mice serum

**FIGURE 8:**
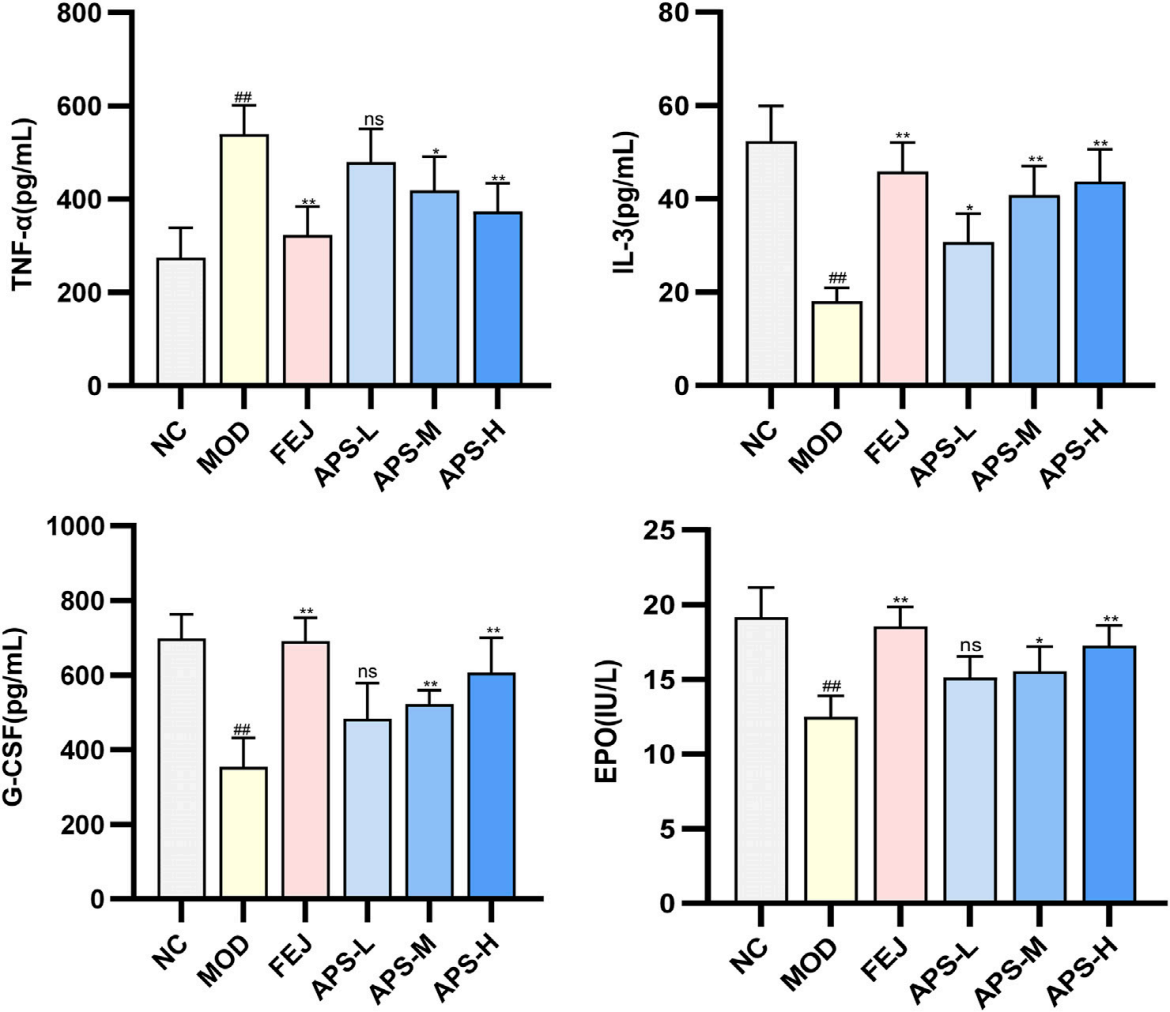
Changes of EPO, G-CSF, IL-3, and TNF-α indexes in BDS mice.

The effect of APS on the serum levels of EPO, G-CSF, IL-3, and TNF-α cytokines in the model mice illustrated in Figure 8, show significantly lower levels of EPO, G-CSF, and IL-3 (*p* < 0.01) and significantly higher level of TNF-α (*p* < 0.01) in mice in the MOD than in the NC group. IL-3 levels and EPO and G-CSF blood levels were significantly higher (*p* < 0.05, *p* < 0.01) in the NC group compared with the MOD group, and TNF-α was significantly lower (*p* < 0.05, *p* < 0.01). This indicated that angelica polysaccharides could dose-dependently promote the levels of hematopoietic factors (EPO, G-CSF, IL-3) and immunomodulatory factors (TNF-α) to be restored to near normal levels.

In this study, APH and CTX were used as inducers to establish a blood-deficient mouse model. After modeling, the mice showed unkempt coat, dull eyes, pale ears and tails, depressed spirit, lethargy, and a significant decrease in the levels of WBC, RBC, HGB, HCT, and PLT in the peripheral blood, which are clinical manifestations of blood deficiency and anemia. Body weight, splenoglandular indices, and the number of peripheral blood cells of the mice were restored to the normal levels after treatment with APS, indicating that APS could effectively reverse blood deficiency syndrome of mice induced by the combination of APH and CTX.

A series of hematopoietic growth factors and co-factors (e.g., G-CSF, M-CSF, EPO, IL-3, IL-6) are involved in the regulation of blood cell formation (Quesenberry, 1986). EPO is an endogenous glycoprotein that stimulates the production of erythrocytes, which promotes the proliferation of erythroid progenitor cells into mature erythrocytes and regulates hematopoiesis in the bone marrow (Zhao et al., 2009). G-CSF and M-CSF enhance the production of hematopoietic progenitors in the bone marrow (Ma et al., 2011). Previous studies have shown that G-CSF and EPO have good synergistic effects on the treatment of anemia (Jadersten et al., 2005). IL-3 is mainly produced by activated CD4^+^ T cells, which plays a very important role in hematopoiesis and immunoregulation, and promotes the differentiation and proliferation of hematopoietic cells, stimulates the growth of a variety of myeloid cells, and thus regulates hematopoiesis (Zhu et al., 2015). TNF-α, a multi-effective pro-inflammatory cytokine and immunomodulatory factor. Inhibits the division of bone marrow stem cells and thus inhibits hematopoiesis, negatively regulating the hematopoietic system (Zhang et al., 2012). Wang J et al. showed that steam-processed Polygonatum sibiricum (SPS) could elevate the levels of hematopoietic cytokines EPO, G-CSF, TNF-α, and IL-6, and regulate key molecules in the JAK1-STAT1 signaling pathway in blood-deficient mice, thus exerting the effects of blood replenishment and immune regulation (Wang et al., 2022). Freeze-dried powder of P. noto ginseng steamed chicken soup (FPSC) can promote the phosphorylation process of JAK2 and STAT5 by activating the JAK2-STAT5 signaling pathway, and then restore the normal secretion of hemostatic regulators, such as EPO, IL-3 and TNF-α levels (Chen et al., 2023). The results of this study showed that after APS administration, the serum levels of EPO, G-CSF, and IL-3 were significantly increased and the serum levels of TNF-α were significantly decreased in mice compared to the MOD group. APS could dose-dependently promote the hematopoietic (EPO, G-CSF, and IL-3) and immunomodulatory (TNF-α) factors in mice of a hemodepletion model to return to normal levels. This suggests that APS may play a role in blood replenishment by enhancing immune function and regulating hematopoietic cytokines.

### 3.9 Analysis of blood-supplementing efficacy *in vitro*

Currently, bioactivity assays are applied in many fields, such as quality evaluation and control of TCM, and evaluation of drug effectiveness or toxicity. Gao et al. established a bioefficacy assay for the antithrombin activity of blood vitality and vessel capsules for quality evaluation and control, by choosing agarose-fibrinogen platelet assay (Gao et al., 2020). Cao et al. established a method to quantitatively determine the anti-platelet aggregation activity of Xiaojin Wan by using the maximum platelet aggregation rate as the evaluation index, and found that Dilong, Wulingyi and Mupeizi might be the key Chinese medicines exerting blood-activating effects in the formula of Xiaojin Wan (Cao et al., 2020). Song et al. used the antagonistic effect of fibrinogen as an evaluation index, and established a bioefficacy assay of rhubarb to evaluate the differences in the efficacy of raw rhubarb, ripe rhubarb and charcoal of rhubarb in activating blood circulation and removing blood stasis (Song et al., 2014).

Drug studies should fulfill safety requirements before exploring drug efficacy. Therefore, the effective concentrations of different angelica polysaccharides were first investigated, and the subsequent effective concentrations were determined to be 64–0.5 μg/mL. The potency AS of the standard hemin solution was set to 1000 U/mg, and the predicted potency of the test product was 1000 U/mg. The standard group was set to have three dose groups of ds1, ds2, and ds3 in the low, medium, and high dosage groups (0.13, 0.26, 0.52 U/mL, respectively). The experimental group was set up with three groups, dt1, dt2, and dt3 (0.01, 0.02, 0.04 U/mL, respectively) of low, medium, and high doses, respectively. Referring to the (3-3) randomized design in the quantitative response parallel line assay of the statistical method for bioassays (General Principle 1431), the measured potency of the test compound was calculated using Formula 1.

**FIGURE 9:**
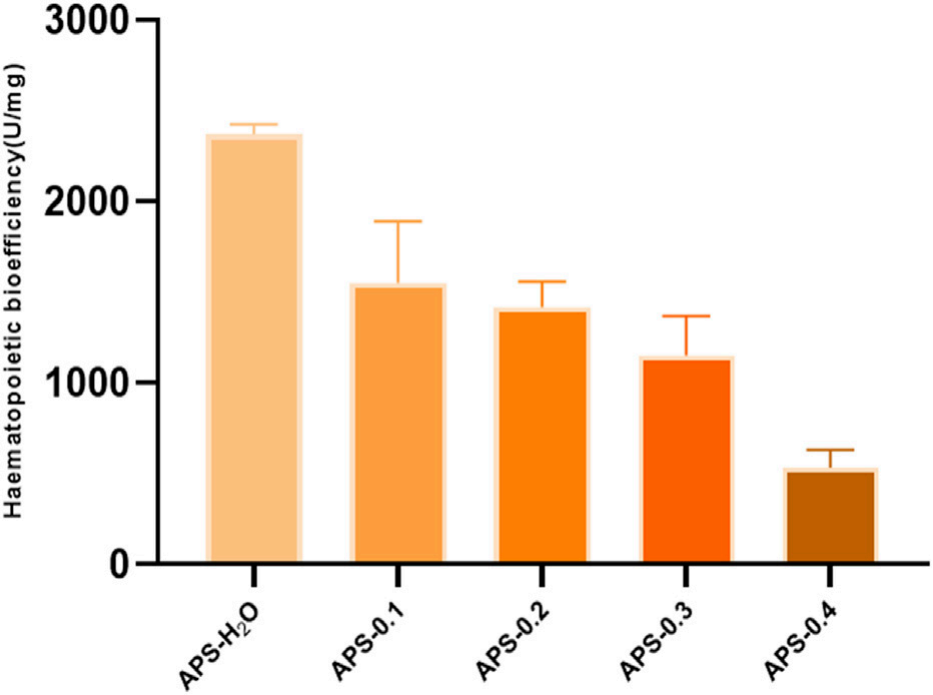
*In vitro* blood supplementation potency of APS-H_2_O, APS-0.1, APS-0.2, APS-0.3 and APS-0.4.

The results of *in vitro* tonic potency determination are shown in Figure 9, and the order of tonic effect of each Angelica polysaccharide was as follows: APS-H_2_O > APS-0.1>APS-0.2>APS-0.3>APS-0.4. Each polysaccharide showed that the higher the polarity of the eluent from the DEAE column, the lower the content of Gal and Ara, and the weaker the tonic effect, i.e., the tonic effect of the neutral Angelica polysaccharides was superior to that of the acidic angelica. The effect of neutral Angelica polysaccharide was better than that of acidic angelica polysaccharide.

## 4 Discussion

This study reveals that APS regulates the proliferation and differentiation of blood cells and promotes the recovery of hematopoietic function in blood-deficient mice by promoting the production of EPO, G-CSF, and IL-3. The reduction of TNF-α content in serum exerted a blood replenishing effect by enhancing the immune function of the body and regulating the hematopoietic function of the bone marrow. There is a strong link between inflammation and BDS. Inflammation affects blood circulation by interfering with the immune system and gut barrier function, and controlling inflammation is important in the prevention and treatment of BDS (Liang et al., 2024). Bioefficacy can better reflect the overall activity and clinical efficacy of TCMs, and a high level of efficacy can be directly quantified using the bioefficacy assay of TCMs. To elucidate the most potent tonifying polysaccharide, we employed our previously developed bioefficacy assay for Angelica sinensis tablets to assess the hemopoietic effects of five distinct polysaccharides derived from Angelica sinensis. Hematopoietic effects were listed in the following order: APS-H_2_O > APS-0.1 > APS-0.2 > APS-0.3 > APS-0.4.

After determining the ranking of the hematopoietic effects of the five APSs, we further analyzed the structure of APS-H_2_O and APS-0.4, to explore possible conformational relationships. In recent years, some scholars have also investigated the polysaccharides with strong hematopoietic activity and their possible structures. Lee et al. (Lee et al., 2012). Isolated four fractions (F1, F2, F3, and F4) from Angelica sinensis by anion-exchange DEAE-Sepharose CL-6B columns, of which F2 had the strongest hematopoietic activity, with the monosaccharide compositions of: Ara (51.82%), Gal (29.96%), GalA (14.80%), Glc (4.78%) and fructose (1.65%). The current study found that the most hematopoietically active APS-H_2_O also contained large amounts of Ara and Gal, which is consistent with Lee et al. Li et al. (Li et al., 2020) isolated and purified a galactose glucan GLP-1 from Ganoderma lucidum, with a monosaccharide composition of Glc:Gal:Man:Fuc = 63.5:26.2:4.9:5.4, which was mainly composed of →6)-β-d-Glcp-(1→, →6) -α-d-Galp-(1→, and →3)-β-d-Glcp-(1→ residues. GLP-1 can increase serum IgA levels, showing a better promotion of hematopoiesis. In this study, we found that APS-H_2_O had the strongest *in vitro* hematopoietic activity, with a monosaccharide composition of Man, Rha, GalA, Glc, Gal, Ara of 0.23:1.00:1.22:0.39:4.83:6.21 Based on the monosaccharide composition, methylation analysis, and nuclear magnetic resonance (NMR) analysis, we hypothesized that APS-H_2_O is an arabinogalactan glycan, with a backbone composed of α −1,3,5-Ara(f), α-1,5- Ara(f), β-1,4-Gal(p) and β-1,4-Gal(p)A, and the two branched chains, β-t-Gal(p) and α-t-Glc(p) connected to α-1,3,5-Ara(f) on the main chain in a (1→3) linkage.

The relationship between blood tonic activity, hepatoprotective effects, and polysaccharide antioxidant activity is strongly correlated. In the case of blood deficiency, blood viscosity is higher than normal, blood flow is slow, and microcirculation perfusion is insufficient, leading to ischemia and hypoxia of body tissues and cells and generation of a large number of free radicals. Therefore, blood tonicity and activation of blood are important means of antioxidant activity and scavenging free radicals. Tao (Zhou, 2018) found that SASP had stronger antioxidant effects than JASP-1A, presumably related to the high Ara content in SASP. Capek P et al. showed that the antioxidant activity of sage polysaccharides enriched with L-Ara and D-Gal was stronger (Capek et al., 2009). Li et al. obtained a total of four polysaccharide fractions from burdock roots, all of which showed high antioxidant capacity, with the neutral polysaccharide ALP-1 having the strongest antioxidant capacity (Li et al., 2021). The Ara content may affect the hepatoprotective effects of ASJP, ASTUP, ASTP, and ASYP, with ASTP having the highest Ara content and the strongest hepatoprotective effect (Hua et al., 2014). Combined with the results of this study, it was hypothesized that Ara content may affect the blood-tonic effect of Angelica polysaccharides, and the specific conformational relationship needs to be further investigated.

While APS-0.4 showed weak tonic activity, the analysis revealed that APS-0.4 contained GalA as the main chain, which may be a pectin structure. Previous studies have found that acidic polysaccharides isolated from Angelica sinensis have good antitumor activity. Cao et al. (Cao et al., 2010). obtained three acidic polysaccharides from Angelica sinensis, and APS-3b and APS-3c showed strong antitumor and immunomodulatory activities *in vivo*, which may be related to their different structural features. Zhang et al. (Zhang et al., 2016). obtained an acidic heteropolysaccharide from Angelica sinensis consisting of GlcA, Glc, Ara, and Gal. An acidic heteropolysaccharide consisting of GlcA, Glc, Ara and Gal with a main frame structure consisting of (1→3)-linked Galp, (1→6)-linked Galp and 2-OMe-(1→6)-linked Galp could effectively inhibit the proliferation of tumor cells. In this study, we hypothesized that the main chain structure of APS-0.4 consists of: α-1,4-GalA, α-1,2-GalA, α-1,4-Gal, and β-1,4-Rha. Pectin has several structural domains, that is, polygalacturonic acid, rhamnogalacturonic acid-I, rhamnogalacturonic acid-II, and xylose-galacturonic acid, and the ratio of the structural domains and branching of the side chains determines the differences in the structure of pectin polysaccharides (Mohnen, 2008). However, in this study, only the main chain structure of APS-0.4 was analyzed. Further analysis of APS-0.4 glycosidic bond conformation and connecting sites, as well as the structure of its side chains, is required to study its activities, such as controlling blood glucose, blood lipids, and antitumor activity.

## 5 Conclusion

In summary, this study demonstrated that APS could elevate levels of positive regulators of hematopoietic system (EPO, G-CSF, IL-3) and reduce levels of negative regulator of hematopoietic system (TNF-α) in the serum of mice, and promote the hematopoiesis of organisms by regulating the levels of positive and negative cytokines. The results of tonic potency assay *in vitro* revealed that APS-H_2_O exhibited the strongest tonic activity. Moreover, it is noteworthy that the hematopoietic effects of the neutral APS were stronger than those of the acidic APS. According to the structural characterization results, APS-H_2_O is an arabinogalactan glycan with a main chain consisting of α-1,3,5-Ara(f), α-1,5- Ara(f), β-1,4-Gal(p), and β-1,4-Gal(p)A. The two branched chains, β-t-Gal(p) and α-t-Glc(p), are connected to the main chain with a (1→3) linkage to the α-1,3,5-Ara(f) connection. In conclusion, APS-H_2_O has significant hematopoietic and myeloprotective effects, and good potential as a drug and nutraceutical for restoring hematopoietic function.

## Data Availability

The original contributions presented in the study are included in the article/Supplementary Material, further inquiries can be directed to the corresponding authors.

## References

[B1] Bai T. K. Tian J. Yan B. (2021). Research progress of clinical application and mechanism of action of seven domestic listed polysaccharide drugs. Mod. Traditional Chin. Med. Materia Medica-World Sci. Technol. 23 (10), 3670–3680. 10.11842/wst.20200903003

[B2] Cao B. Ci Z. M. Xu R. C. Feng B. Xu H. Du X. J. (2020). Quality evaluation of Xiaojin Pills based on antiplatelet aggregation biological potency assay. Chin. Tradit. Herb. Drugs. 10.7501/j.issn.0253-2670.2020.05.022

[B3] Cao P. Sun J. Sullivan M. A. Huang X. Wang H. Zhang Y. (2018). Angelica sinensis polysaccharide protects against acetaminophen-induced acute liver injury and cell death by suppressing oxidative stress and hepatic apoptosis *in vivo* and *in vitro* . Int. J. Biol. Macromol. 111, 1133–1139. 10.1016/j.ijbiomac.2018.01.139 29415408

[B4] Cao W. Li X. Q. Liu L. Wang M. Fan H. T. Li C. (2006). Structural analysis of water-soluble glucans from the root of Angelica sinensis (Oliv.) Diels. Carbohydr. Res. 341 (11), 1870–1877. 10.1016/j.carres.2006.04.017 16682014

[B5] Cao W. Li X. Q. Wang X. Li T. Chen X. Liu S. B. (2010). Characterizations and anti-tumor activities of three acidic polysaccharides from Angelica sinensis (Oliv.) Diels. Int. J. Biol. Macromol. 46 (1), 115–122. 10.1016/j.ijbiomac.2009.11.005 19941888

[B6] Capek P. Machova E. Turjan J. (2009). Scavenging and antioxidant activities of immunomodulating polysaccharides isolated from Salvia officinalis L. Int. J. Biol. Macromol. 44 (1), 75–80. 10.1016/j.ijbiomac.2008.10.007 19014965

[B7] Chen E. L. Li X. X. Wu S. N. Wang M. Niu M. Wang J. B. (2019). Study on quality evaluation of angelica sinensis based on biological titer of activating blood. J. Chin. Med. Mat. 42, 818–821. 10.13863/j.issn1001-4454.2019.04.024

[B8] Chen Z. Chen X. Guo L. Cui X. Qu Y. Yang X. (2023). Effect of different cooking methods on saponin content and hematopoietic effects of Panax notoginseng-steamed chicken on mice. J. Ethnopharmacol. 311, 116434. 10.1016/j.jep.2023.116434 37030555

[B9] Cheng H. N. Neiss G. T. (2012). Solution NMR spectroscopy of food polysaccharides. Polym. Rev. 2 (52), 81–114. 10.1080/15583724.2012.668154

[B10] Gao T. H. Pu J. H. Dong P. Z. Zhang Z. W. Wang T. T. (2020). Establishment of a method to determine biological titer of antithrombin activity in Huoxuetongmai capsule. Chin. Pharm. Bull. 36 (12), 1771–1775. 10.3969/j.issn.1001-1978.2020.12.024

[B11] He Y. Fu C. M. Mao Q. You Y. Hu H. L. Yang L. (2012). Effects of Siwu Decoction with different extracting techniques on hmatopoietic function in mice with blood deficiency. Chin. J. Exp. Tradit. Med. Formulae. 18 (12), 198–200. 10.13422/j.cnki.syfjx.2012.12.061

[B12] Hua Y. Xue W. Zhang M. Wei Y. Ji P. (2014). Metabonomics study on the hepatoprotective effect of polysaccharides from different preparations of Angelica sinensis. J. Ethnopharmacol. 151 (3), 1090–1099. 10.1016/j.jep.2013.12.011 24378353

[B13] Huang D. X. (2005). A discussion on the code of anemia in the international classification, Chin. Med. Rec., 32–33. 10.3969/j.issn.1672-2566.2005.06.019

[B14] Huang G. C. Chen S. Y. Tsai P. W. Ganzon J. G. Lee C. J. Shiah H. S. (2016). Effects of Dang-Gui-Bu-Xue-Tang, an herbal decoction, on iron uptake in iron-deficient anemia. Drug Des. Devel Ther. 10, 949–957. 10.2147/DDDT.S94309 PMC4780732 27041997

[B15] Jadersten M. Montgomery S. M. Dybedal I. Porwit-Macdonald A. Hellstrom-Lindberg E. (2005). Long-term outcome of treatment of anemia in MDS with erythropoietin and G-CSF. Blood 106 (3), 803–811. 10.1182/blood-2004-10-3872 15840690

[B16] Jing P. Song X. Xiong L. Wang B. Wang Y. Wang L. (2023). Angelica sinensis polysaccharides prevents hematopoietic regression in D-Galactose-Induced aging model via attenuation of oxidative stress in hematopoietic microenvironment. Mol. Biol. Rep. 50 (1), 121–132. 10.1007/s11033-022-07898-w 36315330

[B17] Kong L. Yu L. Feng T. Yin X. Liu T. Dong L. (2015). Physicochemical characterization of the polysaccharide from Bletilla striata: effect of drying method. Carbohydr. Polym. 125, 1–8. 10.1016/j.carbpol.2015.02.042 25857953

[B18] Lee J. G. Hsieh W. T. Chen S. U. Chiang B. H. (2012). Hematopoietic and myeloprotective activities of an acidic Angelica sinensis polysaccharide on human CD34+ stem cells. J. Ethnopharmacol. 139 (3), 739–745. 10.1016/j.jep.2011.11.049 22155392

[B19] Li J. Gu F. Cai C. Hu M. Fan L. Hao J. (2020). Purification, structural characterization, and immunomodulatory activity of the polysaccharides from Ganoderma lucidum. Int. J. Biol. Macromol. 143, 806–813. 10.1016/j.ijbiomac.2019.09.141 31715242

[B20] Li L. Qiu Z. Dong H. Ma C. Qiao Y. Zheng Z. (2021). Structural characterization and antioxidant activities of one neutral polysaccharide and three acid polysaccharides from the roots of Arctium lappa L.: a comparison. Int. J. Biol. Macromol. 182, 187–196. 10.1016/j.ijbiomac.2021.03.177 33836197

[B21] Li M. M. Zhang Y. Wu J. Wang K. P. (2020). Polysaccharide from angelica sinensis suppresses inflammation and reverses anemia in complete Freund's adjuvant-induced rats. Curr. Med. Sci. 40 (2), 265–274. 10.1007/s11596-020-2183-3 32337688

[B22] Li W. Y. Li P. Cao W. (2015). A new method to determine the monosaccharides composition in the acidic polysaccharides extracted from the roots of Angelica sinensis (Oliv.) Diels by HPLC. Sci. Technol. Eng. 15 (10), 151–154. 10.3969/j.issn.1671-1815.2015.10.030

[B23] Liang J. Dai W. Liu C. Wen Y. Chen C. Xu Y. (2024). Gingerenone A attenuates ulcerative colitis via targeting IL-17ra to inhibit inflammation and restore intestinal barrier function. Adv. Sci., e2400206. 10.1002/advs.202400206 PMC11267284 38639442

[B47] Liu H. H. Zhang T. Wang M. Yu Y. P. Su L. L. Ji D. (2022). Exploration of a new method for evaluating the quality of Angelica Sinensis based on the determination of blood tonic activity *in vitro* . J. Nanjing Univ. Tradit. Chin. Med., 10.14148/j.issn.1672-0482.2022.1110

[B24] Liu P. J. Hsieh W. T. Huang S. H. Liao H. F. Chiang B. H. (2010). Hematopoietic effect of water-soluble polysaccharides from Angelica sinensis on mice with acute blood loss. Exp. Hematol. 38 (6), 437–445. 10.1016/j.exphem.2010.03.012 20347925

[B25] Liu W. Li W. Sui Y. Li X. Q. Liu C. Jing H. (2019). Structure characterization and anti-leukemia activity of a novel polysaccharide from Angelica sinensis (Oliv.) Diels. Int. J. Biol. Macromol. 121, 161–172. 10.1016/j.ijbiomac.2018.09.213 30290264

[B26] Liu Y. Y. Zhang X. L. Kong Q. Y. Zheng W. H. Yang Y. S. Wang L. (2022). A study on the blood tonifying and immunomodulatory effects of Raw Rehmannia and Mature Rehmannia. J. Chin. Med. Mat. 45 (08), 1853–1856. 10.13863/j.issn1001-4454.2022.08.013

[B27] Ma Z. C. Hong Q. Wang Y. G. Tan H. L. Xiao C. R. Liang Q. D. (2011). Effects of ferulic acid on hematopoietic cell recovery in whole-body gamma irradiated mice. Int. J. Radiat. Biol. 87 (5), 499–505. 10.3109/09553002.2011.548438 21254928

[B28] Mohnen D. (2008). Pectin structure and biosynthesis. Curr. Opin. Plant Biol. 11 (3), 266–277. 10.1016/j.pbi.2008.03.006 18486536

[B29] Ning L. Chen C. X. Jin R. M. Wu Y. P. Zhang H. G. (2002). Effect of components of dang-gui-bu-xue decoction on hematopenia. Chin. J. Chin. Mater Med. 10.3321/j.issn:1001-5302.2002.01.019 12774358

[B30] Niu Y. Xiao H. Wang B. Wang Z. Du K. Wang Y. (2023). Angelica sinensis polysaccharides alleviate the oxidative burden on hematopoietic cells by restoring 5-fluorouracil-induced oxidative damage in perivascular mesenchymal progenitor cells. Pharm. Biol. 61 (1), 768–778. 10.1080/13880209.2023.2207592 37148130 PMC10167876

[B32] Pan S. Jiang L. Wu S. (2018). Stimulating effects of polysaccharide from Angelica sinensis on the nonspecific immunity of white shrimps (Litopenaeus vannamei). Fish. Shellfish Immunol. 74, 170–174. 10.1016/j.fsi.2017.12.067 29305988

[B33] Qian H. L. Pan Z. Q. (2018). Analysis of Traditional Chinese Medicine blood deficiency syndrome and its animal model preparation methods. J. Guangzhou Univ. Tradit. Chin. Med. 35 (01), 176–181. 10.13359/j.cnki.gzxbtcm.2018.01.035

[B34] Quesenberry P. J. (1986). Synergistic hematopoietic growth factors. Int. J. Cell Cloning 4 (1), 3–15. 10.1002/stem.5530040102 3081664

[B35] Song Y. Z. Zeng B. Y. Ren L. Cheng D. Dai C. M. (2014). Biological potency evaluates the effect of processing on promoting blood circulation and removing stasis of rhubarb. Chin. Tradit. Pat. Med. 10.3969/j.issn.1001-1528.2014.09.030

[B36] Wang J. Ge B. Li Z. Guan F. Li F. (2016). Structural analysis and immunoregulation activity comparison of five polysaccharides from Angelica sinensis. Carbohydr. Polym. 140, 6–12. 10.1016/j.carbpol.2015.12.050 26876821

[B37] Wang J. Wang F. Yuan L. Ruan H. Zhu Z. Fan X. (2022). Blood-Enriching effects and immune-regulation mechanism of steam-processed Polygonatum sibiricum polysaccharide in blood deficiency syndrome mice. Front. Immunol. 13, 813676. 10.3389/fimmu.2022.813676 35250989 PMC8892585

[B38] Wang K. P. Zeng F. Liu J. Y. Guo D. Zhang Y. (2011). Inhibitory effect of polysaccharides isolated from Angelica sinensis on hepcidin expression. J. Ethnopharmacol. 134 (3), 944–948. 10.1016/j.jep.2011.02.015 21333724

[B39] Yao H. Y. Wang J. Q. Yin J. Y. Nie S. P. Xie M. Y. (2021). A review of NMR analysis in polysaccharide structure and conformation: progress. challenge and perspective. Food Res. Int. 143, 110290. 10.1016/j.foodres.2021.110290 33992390

[B40] Zhang L. Yu H. L. Yin H. C. Zheng Y. (2011). Blood deficiency analysis of huangdi neijing. Acta Chin. Med. 26 (03), 309–310. 10.16368/j.issn.1674-8999.2011.03.018

[B41] Zhang M. Yin L. Zhang K. Sun W. Yang S. Zhang B. (2012). Response patterns of cytokines/chemokines in two murine strains after irradiation. Cytokine 58 (2), 169–177. 10.1016/j.cyto.2011.12.023 22277799

[B42] Zhang Y. Zhou T. Wang H. Cui Z. Cheng F. Wang K. P. (2016). Structural characterization and *in vitro* antitumor activity of an acidic polysaccharide from Angelica sinensis (Oliv.) Diels. Carbohydr. Polym. 147, 401–408. 10.1016/j.carbpol.2016.04.002 27178946

[B43] Zhao K. J. Li Z. M. Chen X. Y. Dong T. X. Zhan H. Q. (2009). Rationality of mixed preparation of traditional herbal decoction: from the perspective of Danggui buxue Tang. World Sci. Technol. 11 (2), 294–298. 10.1016/S1876-3553(10)60011-7

[B44] Zhou T. (2018). Isolation, structural identification and biological activity of polysaccharides from Angelica sinensis. Doctor, 183. 10.7666/d.D01545795

[B45] Zhu S. J. (2019). Screening of effective parts and separation analysis of chemical composition based on blood nourishing, blood circulation and laxative of Angelica sinensis. Master.

[B46] Zhu Y. L. Zhang J. J. Wang J. X. Yang Z. H. Huang Y. F. Qu S. S. (2015). Comparative study on effects of blood enriching on mouse model of blood deficiency syndrome induced by cyclophosphamide of albiflorin, paeoniflorin on levels of GM-CSF, IL-3 and TNF-α. Chin. J. Chin. Mat. Med. 40 (02), 330–333. 10.4268/cjcmm20150231 26080568

