# Supplementary material for: Structural analysis and blood-enriching effects comparison based on biological potency of Angelica sinensis polysaccharides
Source: Front Pharmacol. 2024 Jun 17;15:1405342. doi: 10.3389/fphar.2024.1405342 (PMC11215113; doi:10.3389/fphar.2024.1405342)
Supplement: Supplementary file 1 [file DataSheet1.docx]

Supplementary Material

## Supplementary Figures 1


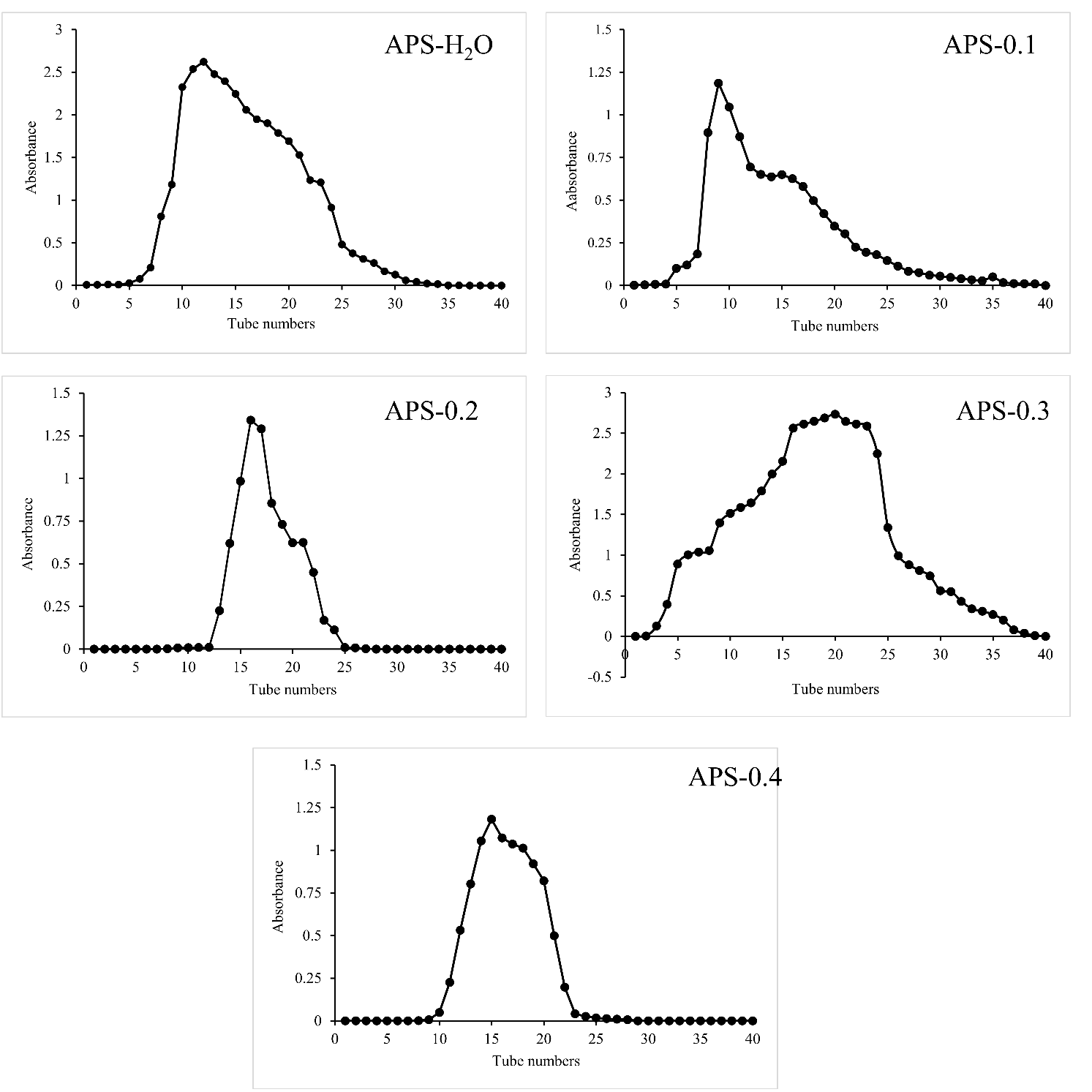


Fig. S1. The elution curve of APS-H_2_O,APS-0.1,APS-0.2,APS-0.3 and APS-0.4 on

Sephadex G-100 column

## Supplementary Figures 2


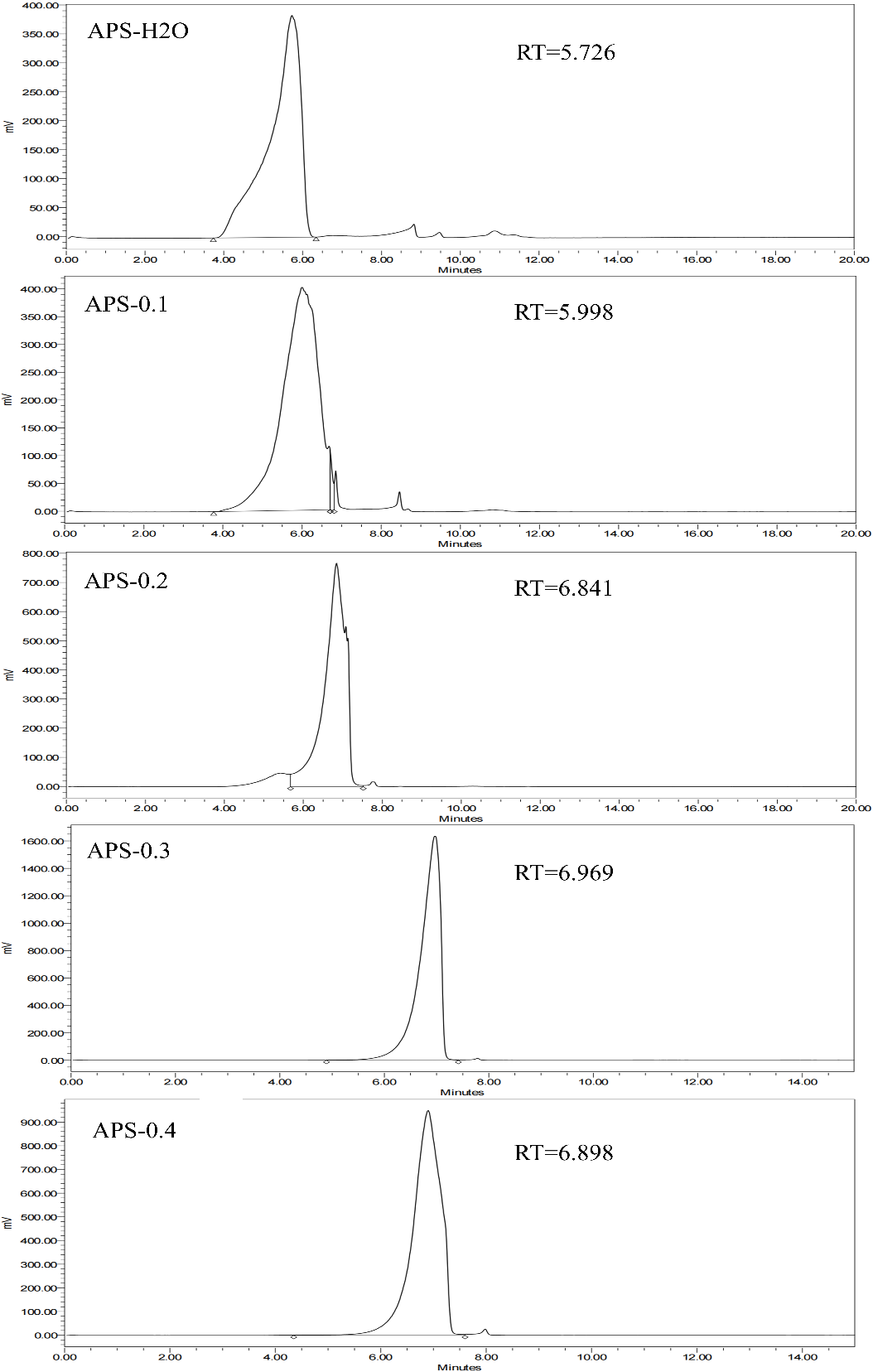


Fig. S2. APS-H2O,APS-0.1,APS-0.2,APS-0.3 and APS-0.4.

## Supplementary Figures 3


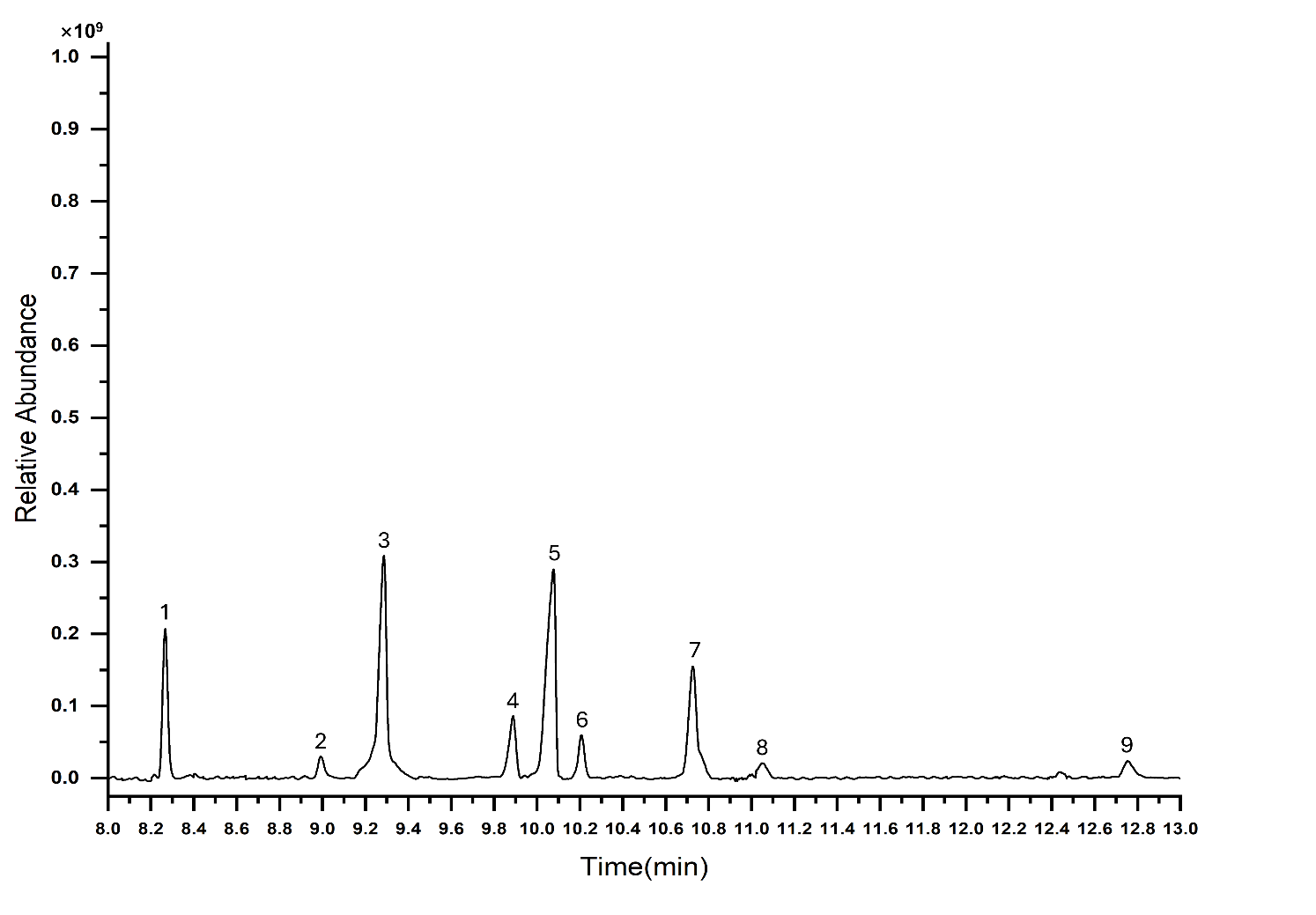
Fig. S3. The GC profiles of methylated alditol acetate of APS-H_2_O. 1. t-Glc(p); 2. 3-Ara(f); 3. 5- Ara(f); 4. t-Gal(p); 5. 3,5-Ara(f); 6. 2,4-Rha(p); 7. 4-Gal(p); 8. 6-Gal(p); 9. 3,6-Glc(p).

GC elution profile of PMAAs derived from APS-H_2_O:

RT：8.266，1,5-Di-O-acetyl-1-deuterio-2,3,4,6-tetra-O-methyl-D-glucitol (t-Glc(p))

RT：8.993，1,3,4-Tri-O-acetyl-1-deuterio-2,5-di-O-methyl-D-arabinitol (3-Ara(f))

RT：9.287，1,4,5-Tri-O-acetyl-1-deuterio-2,3-di-O-methyl-D-arabinitol (5-Ara(f))


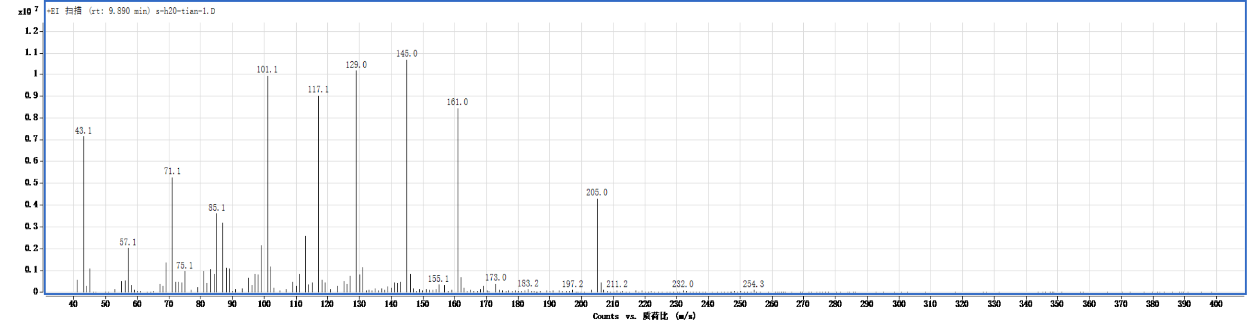
RT：9.889，1,5-Di-O-acetyl-1-deuterio-2,3,4,6-tetra-O-methyl-D-galactitol (t-Gal(p))


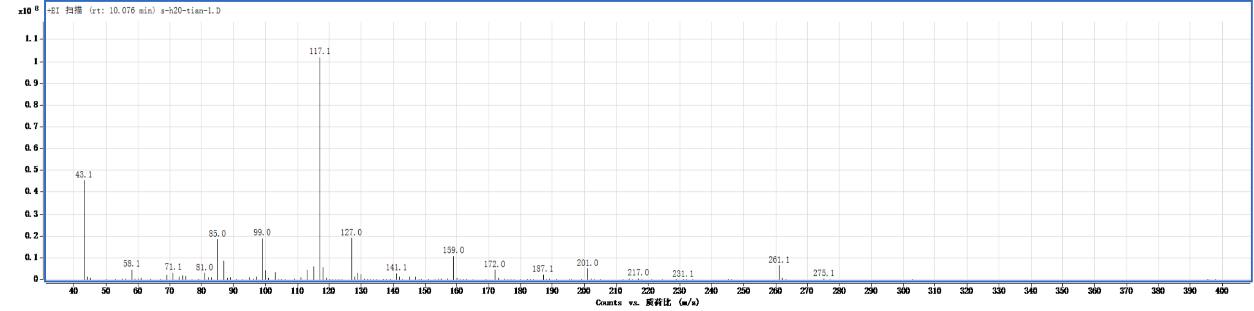
RT：10.078，1,3,4,5-Tetra-O-acetyl-1-deuterio-2-O-methyl-D-arabinitol (3,5-Ara(f))

RT：10.207，1,2,4,5-Tetra-O-acetyl-1-deuterio-6-deoxy-3-O-methyl-L-mannitol (2,4-Rha(p))


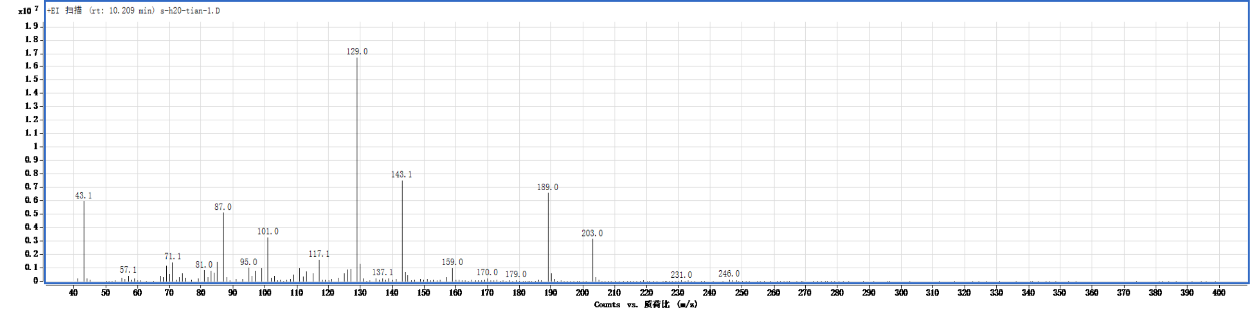

RT：10.729，1,4,5-Tri-O-acetyl-1-deuterio-2,3,6-tri-O-methyl-D-galactitol (4-Gal(p))

RT：11.051，1,5,6-Tri-O-acetyl-1-deuterio-2,3,4-tri-O-methyl-D-glucitol (6-Gal(p))

RT：12.754，1,3,5,6-Tetra-O-acetyl-1-deuterio-2,4-di-O-methyl-D-glucitol (3,6-Glc(p))
